# The Effectiveness of Low-Level LED Light Therapy for Sleep Problems, Psychological Symptoms, and Heart Rate Variability in Shift-Work Nurses: A Randomized Controlled Trial

**DOI:** 10.1155/jonm/6478834

**Published:** 2025-06-17

**Authors:** Yung-Hsuan Liao, Chen-Jei Tai, Jin-Lain Ming, Li-Hwa Lin, Li-Yin Chien

**Affiliations:** ^1^Department of Nursing, Taipei Veterans General Hospital, Taipei, Taiwan; ^2^Institute of Community Health Care, College of Nursing, National Yang Ming Chiao Tung University, Yang-Ming Campus, Taipei, Taiwan; ^3^Dr. Tai's Traditional Chinese Medicine Clinic, Taipei, Taiwan; ^4^College of Medicine, Taipei Medical University, Taipei, Taiwan; ^5^Department of Nursing, College of Nursing, National Yang Ming Chiao Tung University, Yang-Ming Campus, Taipei, Taiwan; ^6^Taipei Municipal Gan-Dau Hospital, Taipei, Taiwan

**Keywords:** heart rate variability, low-level light therapy, psychological symptoms, shift-work nurses, sleep problems

## Abstract

**Background:** Shift-work schedules can cause sleep and psychological problems among nurses, negatively affecting their health and quality of life. This trial examined the effects of low-energy light therapy on sleep, psychological symptoms, and heart rate variability among shift-work nurses.

**Methods:** This randomized controlled trial was conducted from July 2021 to June 2022. The inclusion criteria were nurses with self-reported insomnia who worked in shifts in the last 6 months in a medical center in northern Taiwan. Block randomization was used to assign the study participants to two groups: experimental (*n* = 32) and control (*n* = 32). A portable Meridian Aura Cap equipped with a low-level light-emitting diode was used to provide red and near-infrared light (660 and 850 nm) for 30 min, three times a week for 4 weeks; the control group did not receive any intervention. The Depression Anxiety Stress Scale-21 and Insomnia Severity Index were used to measure psychological symptoms and sleep problems, respectively. Heart rate variability was measured by the ANSWatch.

**Results:** No significant differences were reported in preintervention scores. After the 4-week intervention, the intervention group scored significantly lower in insomnia (4.3 vs. 12.6, respectively; *p* < 0.001), depression (2.5 vs. 7.9, *p* < 0.001), anxiety (3.1 vs. 9.2, *p* < 0.001), and stress (5.6 vs. 12, *p* < 0.001) than those in the controls. No significant differences were observed in heart rate variability between the two groups.

**Conclusion:** Low-level light-emitting diode light therapy improved sleep quality in shift-work nurses with insomnia and alleviated depression, anxiety, and stress symptoms; however, it did not improve heart rate variability, possibly because of the short intervention duration and the ongoing shift-work schedule.

**Implications for Nursing Management:** Employers could consider providing phototherapy for shift nurses to improve their health.

**Trial Registration:** ClinicalTrials.gov identifier: NCT05146596

## 1. Introduction

Nursing involves 24-h continuous patient care, requiring shift work. Shift-work nurses need to adapt to frequently changing circadian rhythms, which cause sleep disturbances and affect hormone secretion and regulation. Shift work increases the risk of cardiovascular disease, dysautonomia, digestive diseases, metabolic syndrome, and obesity, endangering the physical and psychological health of nurses [1–5]. Shift-work disorder is defined as sleep and psychological problems due to shift work [6]. Reportedly, 20%–35% of workers need to work in a shift schedule [5, 7]; of these, 10%–25% suffered from shift-work disorder [7, 8].

Among nurses, shift work is not only related to sleep disturbance but also to psychological symptoms, job stress, and family care responsibilities [2, 9]. A study reported depression in a proportion of 23% of nurses, and their depression rate increased with the increased duration of shift work [10]. Dysautonomia may be caused by illness, sleep problems, stress, anxiety, depression, and emotional problems [11, 12]. It is more prevalent in shift-work nurses than in nurses who do not work on shift. Sleep quality and mental state are associated with heart rate variability (HRV) [13–15].

Low-level light therapy (LLLT) can produce a photobiomodulation (PBM) effect using red light (630–700 nm) and near-red light (700–1100 nm) to irradiate the human body. The proposed mechanism is that photons dissociate inhibitory nitric oxide from enzymes, leading to an increase in electron transport, mitochondrial membrane potential, and ATP production. Another proposed mechanism concerns the activation of light-sensitive ion channels, allowing calcium to enter the cell. After the initial photon absorption events, numerous signaling pathways are activated through reactive oxygen species, cyclic AMP, NO, and Ca^2+^, leading to the activation of transcription factors. These transcription factors can lead to increased expression of genes related to protein synthesis, cell migration, proliferation, anti-inflammatory signaling, antiapoptotic proteins, and antioxidant enzymes [16]. Transcranial PBM occurs when LLLT is placed on the scalp, on the forehead, or intranasally to project light directly to target areas.

One study recruited participants with general anxiety who were asked to self-administer 830 nm light therapy for 20 min a day for 8 weeks; the results showed a significant improvement in anxiety scores and sleep quality [17]. Another study examined the effects of transcranial PBM using 945-nm light-emitting diodes (LEDs) in university students with anxiety and depression. The results showed that the LED transcranial PBM improved brain activity and clinically decreased anxiety and depression [18]. Physical therapy along with PBM (850 and 660 nm) was found to be associated with significantly decreased depression scores compared to physical therapy alone among outpatients with lower back pain and self-reported depression [19]. The effect of separate or combined phototherapy and exercise training was also examined in females suffering from fibromyalgia. Light therapy at 640–905 nm was applied for 300 s on tender points two times per week. The researchers revealed an overlap of therapies in reducing tender point quantity, anxiety, depression, fatigue, and difficulty sleeping; combined therapy produced a more pronounced effect on pain relief and quality of life [20]. In another study examining adults with chronic insomnia, LLLT was applied for 15 min per session, two times a week for 5 weeks. LLLT on acupoints significantly improved insomnia and anxiety compared to the sham LLLT group [21].

Shift-work nurses show sleep difficulty and psychological symptoms, which may be associated with changing circadian rhythms. In addition, the impact of shift work is long-lasting and not limited to workdays [6]. The abovementioned studies show that LLLT alone or combined with other therapies could improve sleep quality and psychological symptoms. Therefore, this therapy may provide a useful treatment. As interventions targeting shift-work nurses have yet to be developed, this study aimed to apply LLLT in shift-work nurses and examine its effectiveness in treating sleep problems, psychological symptoms, and HRV.

## 2. Method

### 2.1. Design and Participants

This study was an unblinded two parallel-group randomized controlled trial. The study participants were shift-work nurses working in a medical center in Taipei, Taiwan, recruited from July 2021 to June 2022. The inclusion criteria were nurses who participated in a 4-week shift work schedule in the last 6 months and had self-reported insomnia with Insomnia Severity Index (ISI) scores ≥ 9. We excluded those with a history of head surgery and who were pregnant. The study participants were recruited through posters, line groups, and the first author's personal network in the study hospital. The eligible participants were informed of the study's purpose and that participation in the study involved committing themselves to 12 measurements in the 4-week study period. All participants signed a written consent form.

Block randomization with a block size of four was used to randomize the study participants into intervention or control groups. Combinations of the assignments were placed in six opaque envelopes. The study participants were given a serial number. Before study initiation, a researcher unsealed the envelopes and assigned participants accordingly.

The required sample size was estimated by G Power 3.1 based on repeated measures with an effect size of 0.2, Type 1 error of 0.05, and power of 0.8, resulting in a required sample size of 42. Since this study involved multiple measures, to account for expected attrition, we aimed to recruit at least 30 participants per group. In total, 64 participants were recruited, with 32 in the intervention group and 32 in the control group. As participants were informed of the commitment needed for participation, all participants completed the study. The study protocol was approved by the Institutional Review Board of the Taipei Veterans General Hospital (IRB no. 2021-06-010C).

### 2.2. Intervention Protocol

A portable Meridian Aura Cap (MAC) (I-Tai, Ministry of Health Medical Devices No. 005077; Top Union Globaltek INC, Hsinchu City, Taiwan) equipped with low-level LED light was used to provide red and near-infrared light (660 and 850 nm) irradiation for 30 min, three times a week for 4 weeks. The MAC was designed using eight soft bands and an optical power maximum of 10 W. Irradiation sites covered the meridians and head acupoints: Baihui (GV20), Shentíng (GV24), Touwei (ST8), Jiaosun (SJ20), Fengchi (GB 20), and Fengfu (DU16). The invention patent number is I702067 (Republic of China, Taiwan). The control group did not receive any intervention.

### 2.3. Measurement

Data were collected using a structured questionnaire and ANSWatch TS0411 (Taiwan Scientific, Xīndiàn, New Taipei, Taiwan). HRV was measured using the ANSWatch. Background variables included sociodemographics (age, sex, educational level, marital status, and children at home), work status (work unit, work years, and shift-work status in the last month and the current month), and use of hypnotics. Background variables were measured at baseline. Psychological symptoms were measured at baseline and every week after the three sessions were completed. Insomnia was measured at baseline and every 2 weeks thereafter.

Psychological symptoms were measured using the Depression Anxiety Stress Scale-21 (DASS-21) [22, 23]. The DASS-21 includes 21 questions with a 4-point Likert scale, with seven questions each for depression, anxiety, and stress. The item sum scores were multiplied by 2 to obtain the final scores (ranging from 0 to 42). Depression scores 0–9 indicated no depression, 10–13 indicated mild depression, 14–20 indicated moderate depression, 21–27 indicated severe depression, and ≥ 28 indicated very severe depression. Anxiety scores 0–7 indicated no anxiety, 8–9 indicated mild anxiety, 10–14 indicated moderate anxiety, 15–19 indicated severe anxiety, and ≥ 20 indicated very severe anxiety. Stress scores 0–14 indicated no stress, 15–18 indicated mild stress, 19–25 indicated moderate stress, 26–33 indicated severe stress, and ≥ 34 indicated very severe stress. The scale was reliable and valid; internal consistency, as measured using Cronbach's alpha, was 0.94, 0.84, and 0.91 for depression, anxiety, and stress, respectively. The scale has been translated into other languages and used in prior research involving nurses [24–26].

Sleep problems were measured using the ISI [27], a seven-item questionnaire asking respondents to rate the nature and symptoms of their sleep problems in the past 2 weeks using a 0–4 Likert-type scale. The scores ranged from 0 to 28; scores 0–7 indicated no sleep problems, 8–14 indicated subthreshold insomnia, 15–21 indicated clinical insomnia with moderate severity, and ≥ 22 indicated clinical insomnia with high severity. The ISI developers performed an initial psychometric study and demonstrated an internal consistency of Cronbach's alpha at 0.74 and found item–total correlations ranging from 0.36 to 0.54. The ISI also correlates significantly with sleep diary reporting [27]. The Chinese version correlated with the Pittsburgh Sleep Quality Index with a correlation coefficient of 0.88 [28]. HRV was measured when sitting on a chair; participants were required to relax and refrain from talking. HRV analysis measures the standard deviation (SD) of normal-to-normal R-wave (SDNN) approved by the Taiwan Ministry of Health and Welfare and the European Union and showed acceptable reliability and validity [29]. The indicators included HRV (25–100 ms), high frequency (HF%; 30−55%), low frequency (LF%; 45−70%), LF/HF (0.8−1.5), and arrhythmia. The number of irregular heartbeats in 5 min exceeding 4 times was defined as arrhythmia. The autonomic nerve ratio is a ratio of LF to HF (LF/HF); the normal value is 0.8–1.5, which represents the balanced state of sympathetic and parasympathetic nerve activity. A ratio outside of this range was defined as dysautonomia; a ratio < 0.8 indicates that the parasympathetic system is more active, while that > 1.5 indicates that the sympathetic system is more active.

### 2.4. Data Analysis

Data were analyzed using IBM SPSS Statistics for Windows, Version 28.0 (IBM Corp, Armonk, NY). Characteristics by intervention or control group were examined using the *t*-test, chi-squared test, or Fisher's exact test. A paired *t*-test or McNemar's X^2^ was used to compare postintervention and preintervention results. Generalized estimation equations (GEEs) were used to analyze time-dependent outcomes, given that confounders were adjusted.

## 3. Results

The baseline characteristics of the intervention and control groups are presented in Table 1. The majority of the participants (87.5%) were females; all participants had a university or postgraduate degree, and more than 70% worked in medical wards. The intervention group was younger than the control group (26.19 vs. 29.50, respectively, *p*=0.01). A higher proportion of the intervention group had worked for ≤ 5 years compared to the controls (80.73% vs. 56.24%, respectively), were unmarried (93.75% vs. 81.25%), and did not have children (100% vs. 87.5%). Shift-work status in the past month was mostly day shift (46.86% and 56.25% for intervention and controls, respectively), followed by night (28.14% and 21.88% for intervention and controls, respectively) and evening shifts (25% and 21.88% for intervention and controls, respectively). For the current month, slightly more nurses were on the day shift than on the night shift (44.76% and 40.61% for the day shift for intervention and controls, respectively; 40.61% for the night shift in both groups), with the least being on evening shift (15.63% and 18.78% for intervention and controls, respectively). Only four participants used sleep pills in the past month (three in the intervention group and one in the control group). No statistically significant differences were found in the distribution of characteristics between groups except for age. In addition, there were no significant differences in preintervention severity of insomnia and psychological symptoms between the intervention and control groups.

Tables 2 and 3 present the crude and adjusted effects of LLLT on psychological symptoms, respectively. No significant differences were reported in preintervention insomnia, depression, anxiety, and stress scores between the intervention and control groups (Table 2). After the 4-week LLLT intervention, the intervention group showed greater improvement in insomnia (mean difference: −8.34, *p* < 0.001), depression (−5.44, *p* < 0.001), anxiety (−6.13, *p* < 0.001), and stress (−7.5, *p* < 0.001) than those in controls. For anxiety and stress, significant differences between the intervention and control groups were observed after 1 week of LLLT, and after 2 weeks for depression and insomnia. Differences between the intervention and control groups increased with time receiving LLLT.

In Table 2, we additionally presented the proportion of participants with insomnia and psychological symptoms and compared differences in proportions between the intervention and control groups. The intervention group showed a lower rate of preintervention depression, anxiety, and stress than the controls. The differences were statistically significant for preintervention anxiety but not for depression and stress. Greater differences in proportions with the symptoms were observed after the 4-week LLLT intervention. However, the differences appeared to be more salient when comparing mean scores than proportions. Therefore, we decided to proceed with the analysis with scores rather than proportions.

As significant differences in age and probable differences in preintervention psychological state were found between the intervention and control groups, GEE was used to yield age- and preintervention psychological state-adjusted effects (Table 3). The adjusted results showed that LLLT was effective in treating insomnia after 2 weeks (*ß* = −3.87, *p* < 0.001), with the effect increasing after 4 weeks (*ß* = −7.88, *p* < 0.001). For depression (ß = 0.44, −1.19, −2.44, and −2.69 for weeks 1–4), anxiety (*ß* = −2.19, −2.50, −2.25, and −4.44), and stress (*ß* = −1.94, −3.44, −3.31, and −5.19), the effect appeared to increase with time. A significant effect appeared after 1 week of LLLT for anxiety, 2 weeks for stress, and 4 weeks for depression.

The results of HRV indicators, including arrhythmia, HRV, and LF/HF, did not differ significantly between the intervention and control groups after the 4-week LLLT. Approximately 30% of study nurses had arrhythmia pretests (31% for both groups). After completion of the 4-week LLLT, the proportion of study nurses with arrhythmia was 28% in the intervention group and 37% in the control group (*p*=0.60). Baseline HRV was not significantly different (*p*=0.74) between the intervention (mean: 50.25, SD: 15.51) and control groups (mean: 47, SD: 17) before the LLLT intervention. After completion of the 4-week LLLT, the posttest HRV was not significantly different (*p*=0.08) between the intervention group (mean: 54.56, SD: 17.49) and controls (mean: 47.28, SD: 16.04). As for LF/HF, the baseline ratio was significantly lower (*p*=0.01) for the intervention group (0.97 ± 0.91) than for the controls (1.67 ± 1.23). After completion of the 4-week LLLT, the posttest LF/HF was not significantly different (*p*=0.09) between the intervention group (1.21 ± 1.28) and controls (1.71 ± 1.66).

Approximately 66% of study nurses had dysautonomia pretest (control group: 63%, intervention group: 69%). After completion of the 4-week LLLT, the proportion of study nurses with dysautonomia was 72% in the intervention group and 75% in the control group (*p*=1.00).

## 4. Discussion

This study found that LLLT intervention three times a week for 4 weeks was effective in treating insomnia, depression, anxiety, and stress symptoms among shift-work nurses with self-reported insomnia. The study nurses were on a 1-month fixed shift (the same shift schedule in a month); as a result, the intervention time was set at 4 weeks to decrease the effect of the transition between day, evening, and night work. The light waves, source, irradiation times, and duration differ across previous studies, limiting the comparability with the current study. Despite this, the present study agreed with previous studies in showing the effectiveness of LLLT in improving sleep problems and psychological symptoms [17–19, 21, 30]. In addition to the overall effectiveness, the effect of LLLT appeared after 1 week for anxiety and stress and after 2 weeks for insomnia and depression, growing with time. The results suggest that the effect of LLLT could be cumulative. However, given that our study only lasted for 4 weeks, additional studies are needed to examine long-term implications.

The full mechanisms of LLLT remain under investigation. Nevertheless, transcranial PBM using red or near-infrared light can penetrate the skull, enhance cytochrome c oxidase activity, and boost mitochondrial metabolism [31]. In cell culture studies, LLLT has been found to promote cellular metabolism, enhance blood circulation, and support the regeneration and repair of nerve cells and tissues [32]. LLLT improves the function and survival of neurons in the brain by stimulating mitochondrial activity and activating various excitatory and protective pathways, influencing neurotrophic factor expression and improving emotional functions [33]. From a TCM perspective, stimulating meridian acupoints may restore qi flow and internal energy, contributing to improvements in insomnia and psychological symptoms [34]. Together, these pathways may explain the observed benefits of insomnia, depression, anxiety, and stress in the study.

The LLLT intervention was not effective in altering HRV indicators, though the prevalence of arrhythmia was 31% preintervention and 28% postintervention. The prevalence of dysautonomia was 69% preintervention and 72% postintervention. Prior research suggests that shift work is related to circadian rhythm irregularity that can lead to cardiac dysautonomia [1–3]. The high prevalence of arrhythmia and dysautonomia among shift-work nurses indicates a need to direct resources to alleviate poor health conditions.

Past studies have shown that nurses' shift work and circadian rhythm disorders affect heart rhythm variability and cause abnormal indicators [13–15, 35]. Various indicators of heart rhythm variability are affected by physiological status and environment, such as circadian rhythm, drugs, living habits, menstrual cycle, menopause, aging, exercise, essential oil smell, sleep, psychological symptoms, stress, and disease status [11, 12, 36, 37]. The current study found that, after 4 weeks of intervention, no statistically significant differences were found in HRV-related indicators. Previous studies have not used HRV as an outcome measure for LLLT. The lack of effect on HRV indicators may be due to the short duration of the intervention in this study; as such, future studies with longer interventions are needed.

Due to their work characteristics, job stress, and shift-work schedules, nurses have a high risk of psychological stress and chronic diseases [2, 4, 5, 38]. Intervention is needed to promote the health of nurses. Our study results suggest that hospitals could set up health promotion centers in the hospital incorporating LLLT for healthcare workers, which is in line with the Health Promoting Hospital Initiatives [39]. The advantage of LLLT includes its noninvasiveness and lack of complications. The device used in this study is easy to use and could also be used at home by nurses to improve sleep quality and psychological symptoms.

### 4.1. Limitations

Though we used random assignment, the intervention group was significantly younger than the control group. Besides, the intervention group showed lower pretest scores in depression, anxiety, and stress, though not statistically significant. Therefore, we applied GEE to adjust for age and preintervention states. The study nurses were from a single medical center, and the sample size was small. Future studies with a heterogeneous population and larger sample sizes are needed. The shift work in this study was based on a fixed and regular 8-h shift. Further studies are also needed to examine and compare LLLT's effectiveness across different shift-work schedules and patterns.

## 5. Conclusion

LLLT three times a week for 4 weeks improved sleep quality in shift-work nurses with insomnia and alleviated depression, anxiety, and stress symptoms; however, it did not improve HRV. This is a single-center study with a relatively small sample size, while the results are promising, studies with larger sample sizes and at different hospital settings are needed to further evaluate the effects before recommending widespread implementation.

## Figures and Tables

**Table 1:** Characteristics of the study participants (*n* = 64).

Variables	Control (*n* = 32)	Intervention (*n* = 32)	*t*/*χ*^2^	*p*
	*n* (%)	*n* (%)		
Sex			—	1.000
Male	4 (12.5%)	4 (12.5%)		
Female	28 (87.5%)	28 (87.5%)		
Age, M (SD)	29.50 ± 5.20	26.19 ± 5.13	2.565	^∗^0.013
Educational level			—	0.672
University	28 (87.5%)	30 (93.75%)		
Postgraduate	4 (12.5%)	2 (6.25%)		
Using sleep pills in the past month			—	0.613
No	31 (96.87%)	29 (90.62%)		
Yes	1 (3.13%)	3 (9.38%)		
Unit			1.634	0.442
Medical ward	26 (81.25%)	20 (62.5%)		
Surgical ward	6 (18.75%)	8 (25%)		
Mixed ward	0 (0%)	4 (12.5%)		
Working years			5.602	0.133
0-1	5 (15.63%)	11 (34.38%)		
2–5	13 (40.61%)	15 (46.86%)		
6–10	5 (15.63%)	2 (6.25%)		
> 10	9 (18.75%)	4 (6.25%)		
Current marital status			—	0.257
Unmarried	26 (81.25%)	30 (93.75%)		
Married	6 (18.75%)	2 (6.25%)		
Having children			—	0.113
No	28 (87.50%)	32 (100%)		
Yes	4 (12.50%)	0 (0%)		
Shift status in the past month			0.589	0.745
Day shift	18 (56.25%)	15 (46.86%)		
Evening shift	7 (21.875%)	8 (25%)		
Night shift	7 (21.875%)	9 (28.14%)		
Shift status this month			0.128	0.938
Day shift	13 (40.61%)	14 (44.76%)		
Evening shift	6 (18.78%)	5 (15.63%)		
Night shift	13 (40.61%)	13 (40.61%)		
Insomnia			1.024	0.599
Mild	20 (63%)	21 (66%)		
Moderate	11 (34%)	11 (34%)		
Severe	1 (3%)	0 (0%)		
Depression			3.532	0.473
Mild	5 (16%)	4 (12%)		
Moderate	6 (19%)	6 (19%)		
Severe	1 (3%)	1 (3%)		
Extremely severe	3 (9%)	0 (0%)		
Anxiety			7.002	0.136
Mild	5 (16%)	2 (6%)		
Moderate	14 (44%)	7 (22%)		
Severe	3 (9%)	4 (13%)		
Extremely severe	2 (6%)	2 (6%)		
Stress			1.266	0.737
Mild	8 (25%)	5 (16%)		
Moderate	4 (13%)	3 (9%)		
Severe	2 (6%)	3 (9%)		
Extremely severe	0 (0%)	0 (0%)		

*Note:* Insomnia was determined by ISI scores (mild: 8–14, moderate: 15–21, and severe: ≥ 22). Depression, anxiety, and stress were determined by DASS-21 (depression: mild 10–13, moderate 14–20, severe 21–27, and extremely severe ≥ 28; anxiety: mild 8-9, moderate 10–14, severe 15–19, and extremely severe ≥ 20; stress: mild 15–18, moderate 19–25, severe 26–33, and extremely severe ≥ 34).

^∗^ *p* < 0.005.

**Table 2:** Mean scores and proportions with sleep quality and psychological symptoms by intervention and control status.

	Control (*n* = 32)			Intervention (*n* = 32)			Mean difference, *p*	Differences in % with the symptom, *p*
	Mean	SD	*n* (%) with the symptom	Mean	SD	*n* (%) with the symptom		
*Sleep measures*								
Insomnia								
Pretest	13.81	4.04	32 (100%)	13.34	3.01	32 (100%)	−0.47, 0.600	0, —
2^nd^ week	12.44	4.29	30 (94%)	8.09	3.48	19 (59%)	−4.35, ^∗^< 0.001	−35%, ^∗^0.002
4^th^ week	12.59	4.79	30 (94%)	4.25	3.3	5 (16%)	−8.34, ^∗^< 0.001	−78%, ^∗^0.001

*Psychological measures*								
Depression								
Pretest	10.44	8.03	15 (47%)	7.69	6.18	11 (34%)	−2.75, 0.130	−13%, 0.446
1^st^ week	7.38	6.86	11 (34%)	5.06	5.1	9 (28%)	−2.32, 0.131	−6%, 0.788
2^nd^ week	8.19	7.54	12 (38%)	4.19	4.58	4 (13%)	−4, ^∗^0.013	−25%, ^∗^0.041
3^rd^ week	8.13	7.6	11 (34%)	3.13	3.63	2 (6%)	−5, ^∗^0.002	−28%, ^∗^0.011
4^th^ week	7.94	7.51	13 (41%)	2.5	3.7	3 (9%)	−5.44, ^∗^0.001	−32%, ^∗^0.008
Anxiety								
Pretest	10.06	5.67	24 (75%)	8.38	6.71	15 (47%)	−1.68, 0.281	−28%, ^∗^0.039
1^st^ week	9.38	5.55	19 (59%)	5.5	6.03	9 (28%)	−3.88, ^∗^0.010	−31%, ^∗^0.023
2^nd^ week	9.06	5.81	19 (59%)	4.75	4.68	8 (25%)	−4.31, ^∗^0.002	−34%, ^∗^0.011
3^rd^ week	8.63	6.51	17 (53%)	4.63	5.24	7 (22%)	−4, ^∗^0.009	−31%, ^∗^0.019
4^th^ week	9.19	5.92	18 (56%)	3.06	4.4	4 (13%)	−6.13, ^∗^< 0.001	−43%, ^∗^< 0.001
Stress								
Pretest	14.5	5.99	14 (44%)	12.19	8.21	11 (34%)	−2.31, 0.246	−10%, 0.609
1^st^ week	13	6.83	9 (28%)	8.75	6.85	7 (22%)	−4.25, ^∗^0.016	−6%, 0.774
2^nd^ week	12.69	7.63	10 (31%)	6.94	6.45	4 (13%)	−5.75, ^∗^0.002	−18%, 0.129
3^rd^ week	11.94	7.28	9 (28%)	6.31	5.99	2 (6%)	−5.63, ^∗^0.001	−22%, ^∗^0.043
4^th^ week	12	7.26	10 (31%)	4.5	5.61	3 (9%)	−7.5, ^∗^< 0.001	−22%, 0.060

^∗^ *p* < 0.05.

**Table 3:** Generalized estimation equation model of the effect of low-level LED light therapy on insomnia, depression, anxiety, and stress.

Variables	Insomnia		*p*	Depression		*p*	Anxiety		*p*	Stress		*p*
	B	SE		B	SE		B	SE		B	SE	
Intercept	13.98	2.22	< 0.001	8.20	4.72	0.082	5.80	3.46	0.094	12.74	3.77	^∗^< 0.001
Intervention group^†^	−0.48	0.91	0.593	−2.50	1.88	0.184	−1.21	1.52	0.426	−2.11	1.79	0.237
Time (4^th^ week)^‡^	−1.21	0.56	^∗^0.030	−2.50	0.86	^∗^0.004	−0.87	0.90	0.332	−2.50	1.10	^∗^0.023
Time (3^rd^ week)^‡^	NA			−2.13	1.00	^∗^0.034	−1.50	0.99	0.131	−2.56	1.00	^∗^0.010
Time (2^nd^ week)^‡^	−1.37	0.47	^∗^0.003	−2.31	0.94	^∗^0.014	−1.13	0.85	0.184	−1.81	1.11	0.103
Time (1^st^ week)^‡^	NA			−3.06	0.85	^∗^< 0.001	−0.69	0.74	0.351	−1.50	0.77	0.052
Intervention × time (4^th^)^§^	−7.88	0.84	^∗^< 0.001	−2.69	1.34	^∗^0.045	−4.44	1.34	^∗^0.001	−5.19	1.59	^∗^0.001
Intervention × time (3^rd^)^§^	NA			−2.44	1.30	0.060	−2.25	1.28	0.080	−3.31	1.43	^∗^0.020
Intervention × time (2^nd^)^§^	−3.87	0.77	^∗^< 0.001	−1.19	1.27	0.349	−2.50	1.16	^∗^0.032	−3.44	1.47	^∗^0.019
Intervention × time (1st)^§^	NA			0.44	1.08	0.687	−2.19	1.03	^∗^0.033	−1.94	1.33	0.145
Age	−0.006	0.07	0.932	0.08	0.15	0.607	0.14	0.11	0.205	0.06	0.12	0.622

^†^Reference group: Control group.

^‡^Reference group: Time (pretest).

^§^Reference group: Group (control) × time (pretest).

^∗^ *p* < 0.05.

## Data Availability

The data that support the findings of this study are available from the corresponding author upon reasonable request.
